# Outcomes of a care bundle strategy at a dedicated private surgical center for idiopathic normal pressure hydrocephalus

**DOI:** 10.1007/s00701-026-07075-z

**Published:** 2026-10-22

**Authors:** Daniel de Wilde, Victor E. Staartjes, Rafael Holmgren, Erik Edström, Adrian Elmi-Terander

**Affiliations:** 1 Machine Intelligence in Clinical Neuroscience (MICN) & Microsurgical Neuroanatomy Laboratory, Department of Neurosurgery, Clinical Neuroscience Center, University Hospital Zurich, University of Zurich, Zurich, Switzerland; 2 Capio Spine Center Stockholm, Löwenströmska Hospital, Upplands Väsby, Sweden; 3 Department of Clinical Neuroscience, Karolinska Institutet, Stockholm, Sweden; 4 Department of Biomedical and Clinical Sciences, Linköping University, Linköping, Sweden; 5 Department of Surgical Sciences, Uppsala University, Uppsala, Sweden; 6 Department of Medical Sciences, Örebro University, Örebro, Sweden

**Keywords:** Hydrocephalus, Normal pressure, Ventriculoperitoneal shunt, Clinical protocols, Care bundle, Patient satisfaction

## Abstract

**Background:**

Idiopathic normal pressure hydrocephalus (iNPH) is a prevalent and surgically treatable condition in the elderly. Ventriculoperitoneal (VP) shunting is the standard treatment, yet outcomes may vary depending on surgical expertise and care delivery models. This study evaluates outcomes of a care bundle strategy for iNPH at a private healthcare provider under public contract.

**Methods:**

We performed a retrospective analysis of a prospectively collected cohort of consecutive patients undergoing VP shunt placement for iNPH at a private hospital between November 2024 and November 2025. All procedures were performed by two senior consultant neurosurgeons adhering to a care bundle strategy. Clinical outcomes and patient-reported experience measures (PREM) were assessed at one month postoperatively.

**Results:**

Eighty patients were included, with 100% follow-up for clinical outcomes and 83% response (*n* = 60) to PREMs. Mean time from referral to surgery was 28.5 ± 15 days. Overall clinical status improved in 93% of patients, with a gait improvement in 88%, and improvement rates of 24% and 29% for continence and cognition, respectively. No reoperations were required within the timeframe of the study. Patient satisfaction was high across all domains, with a mean likelihood-to-recommend score of 9.8 ± 0.69.

**Conclusion:**

VP shunt placement within a consultant-led care bundle pathway at a private surgical center was associated with high patient-reported efficacy, low complication and reoperation rates, and excellent patient satisfaction. These findings support the feasibility and safety of dedicated iNPH care bundle models outside traditional academic centers to improve timely access to treatment.

## Introduction

Idiopathic normal pressure hydrocephalus (iNPH) is a clinical syndrome characterized by communicating hydrocephalus despite normal or intermittently elevated cerebrospinal fluid (CSF) pressure. It is classically defined by the triad of gait disturbance, cognitive impairment, and urinary incontinence [1, 12]. The condition affects the elderly population, increasing in incidence from the age of 65 years, and its prevalence is expected to increase along with the aging population [9, 32].

The standard treatment of iNPH consists of cerebrospinal fluid diversion, most commonly through ventriculoperitoneal (VP) shunting. Less commonly used alternatives include lumboperitoneal shunting, ventriculoatrial shunting, endoscopic third ventriculostomy, and choroid plexus coagulation [3, 17, 33]. Surgical intervention has been shown to result in symptomatic improvement in most patients [6, 33]. However, complication and revision rates remain non-negligible, with prior studies reporting complication rates in the range of approximately 8–20% and reoperation rates approaching 10% [25].

CSF diversion procedures are frequently regarded as technically straightforward and are therefore often performed by less experienced surgeons as part of neurosurgical training [26]. While this paradigm is well established in clinical practice, it raises the question of whether outcomes may be influenced by surgeon experience and the degree of standardization in care delivery. Despite the high prevalence of iNPH and the widespread use of shunt procedures, there is limited evidence specifically examining outcomes when these procedures are performed by senior consultants in highly standardized care settings. Furthermore, recent national Swedish registry data consistently show prolonged waiting times for surgery, emphasizing a growing need for scalable and accessible iNPH care pathways [28]. Previous research has demonstrated that prolonged waiting time (i.e., 3 months) can negatively affect clinical outcomes in patients with iNPH [7].

To provide iNPH patients with timely access to surgical treatment and improved outcomes, a more accessible and standardized iNPH center was established at a private healthcare provider under public contract**.** To ensure safety and surgical quality, all procedures were performed by senior neurosurgical consultants. A care bundle strategy was chosen to standardize the entire patient pathway, from referral assessment to postoperative follow-up, with the aim of improving consistency of care [10, 14, 21], following the principles outlined by Borgert et al. [4].

The present study aims to evaluate the clinical outcomes and patient-reported experience measures associated with this dedicated care model.

## Methods

### Study design

The present study is a retrospective analysis of a prospectively maintained cohort of patients undergoing surgical treatment for iNPH. The surgical procedure was performed at a newly established, dedicated NPH center managed by a private healthcare provider (Capio Spine Center Stockholm, Löwenströmska Hospital) working under public contract. A care bundle strategy was employed in setting up the center and was implemented for all patients in the study. The study was conducted in accordance with the Strengthening the Reporting of Observational Studies in Epidemiology (STROBE) guidelines. All adult patients who underwent VP-shunt implantation for NPH between November 2024 and November 2025 were eligible for inclusion. All procedures were performed by two senior consultant neurosurgeons (E.E., A.E.T.) each with more than 20 years of experience.

The study center had extensive experience in elective spinal surgery but no prior experience in neurosurgical procedures. Prior to the initiation of neurosurgical activity, a structured educational program was undertaken across all staff categories involved in the patient pathway, from outpatient clinic personnel to operating theatre and ward staff, comprising lectures, video demonstrations, and case-based presentations. A patient information brochure was developed based on existing published patient materials, adapted to the specific setting and workflow of the center. The surgical procedure and perioperative workflow were rehearsed through repeated simulation exercises, allowing each step of the pathway to be tested, refined, and standardized prior to the treatment of the first patient. The surgical technique itself was developed and optimized based on the combined neurosurgical and hydrocephalus-specific experience of the two consultants.

### Patient population

The Swedish healthcare system mandates that no patient, having been referred from a general practitioner, should wait more than 90 days for an appointment with a specialist and no more than 90 days waiting for a surgical procedure. The patients in this study had been referred to the Department of Neurology at a Swedish University hospital (Linköping) and diagnosed with iNPH based on a multi-professional team assessment. Neuroradiologists assessed the patients' computed tomography examinations according to the iNPH Radscale for the probability of iNPH [15]. All eligible patients were examined with MRI to rule out hydrocephalus of other causes such as aqueductal stenosis. All patients were assessed by a senior neurologist subspecialized in motor disorders, physiotherapist for motor symptoms and occupational therapist for cognitive skills. Lumbar puncture was performed for assessment of lumbar opening pressure and CSF-markers of neurodegenerative disorders. No tap test was performed as this routine was discontinued at the institution in 2022 due to its poor negative predictive value [20]. Patient findings were discussed in the multi-professional team, including neurosurgeons, and iNPH diagnosis was established according to iNPH guidelines [19]. Eligibility for shunting was decided at the same conference and the patients then met with, were informed by and offered shunt placement surgery by a neurosurgeon. Moreover, the patients had met with, been informed by, and been offered shunt placement surgery by a neurosurgeon. The majority of the patients were referrals, based on failure to provide surgery within the mandated waiting time, from neurosurgical departments at a university hospital in Sweden. Four patients were referred after diagnosis at a private neurological clinic. Referrals from neurosurgical departments were reviewed as soon as possible upon arrival, and patients were contacted by phone to ensure that they were well informed and consented to the procedure and to identify potential risks or practical obstacles that could have been lacking in the referral information. Patients considered suitable candidates for surgical treatment at the center were scheduled accordingly. To ensure patient safety, patients with severe comorbidities or in very poor general health were declined treatment at the center. Relevant conditions included heart failure, renal failure grade 3 or above, a requirement for full-time assistance, or being bedridden. Of the patients referred from a neurosurgical department, 4 were declined. Among these, two were deemed unsuitable due to substantial comorbidity, one had epilepsy requiring valproate treatment, one had severe renal insufficiency.

## Treatment protocol

A care bundle strategy was used for all patients to ensure consistency in care delivery (Table 1) [13]. All predefined components of the care bundle were implemented in all patients, and no deviations from the protocol were documented. Prior to admission, all patients underwent a structured preoperative physiotherapy assessment to evaluate functional status and activities of daily living, as well as to identify factors potentially affecting the surgical procedure or postoperative care. Preoperative cranial computed tomography (CT) imaging was performed in all cases.

**Table 1:** Overview of the standardized iNPH care bundle from referral through postoperative follow-up

Care pathway	Key issue addressed	Care bundle solution
Referral & Triage	Delayed access and inconsistent referral processing	Rapid referral review, patient contact, eligibility screening, prioritized scheduling
MDT Assessment	Variation in diagnostic assessment and treatment selection	Neurology, PT/OT and neurosurgical assessment with standardized MDT decision-making
Preoperative Preparation	Incomplete functional assessment and patient information	Standardized functional assessment, CT imaging, patient information booklet
VP Shunt Surgery	Variation in surgical expertise and technique	Two senior consultants, standardized surgical technique, standardized valve settings
Infection Prevention	Surgical contamination and shunt infection	Hair clipping, skin preparation, double gloves, protocolized glove changes, cranial/abdominal field separation
Quality Assurance	Catheter malposition and perioperative complications	Routine early cranial and abdominal CT, verification of catheter position, exclusion of hemorrhage before discharge
Follow-up & Monitoring	Inconsistent postoperative monitoring and outcome assessment	Standardized PROM/PREM assessment, 6–8 week CT, registry reporting and defined long-term follow-up responsibilities

MDT: multidisciplinary team; PT: physical therapy, OT: occupational therapy; CT: computed tomography; PROM: patient-reported outcome; PREM: patient-reported experience measure

The surgical procedure consisted of ventriculoperitoneal shunt implantation using a standardized technique where one surgeon performed the cranial part and another surgeon performed the abdominal part of the procedure using their separate sets of instruments. All patients were shaved using hair trimmers on the whole implantation side, and the skin was meticulously cleaned using a Descutan sponge. Dual-layer latex-free indicator gloves were used, and the outer gloves were replaced with new gloves before any contact with the shunt valve or tubing to ensure sterile handling. All surgical drapes, which can transfer microfibers, were covered with plastic sheets where contact with the shunt was a possibility.

Cranially, a straight incision was placed to allow a right-sided (if not contraindicated) burr hole at Kocher’s point. A second incision was placed cranial and posterior to the ear to ensure sufficient space for a pocket extending towards the mastoid process. In the abdomen the incision was placed over the ipsilateral rectus abdominis muscle below the arcus costae. A more cranial positioning of this incision was favoured to minimize the risk of catheter extrusion. For the same reason, tunnelling, from cranial to abdominal, was performed to ensure a passage deep to the outer fascia of the rectus abdominis. All incisions were sutured with resorbable sutures, and skin staples were applied after renewed sterile cleaning of the site. All suture lines were sprayed with Spray Patch before final patch placement. The standardized incision locations were used in all patients, with left-sided placement used when previous surgery, implanted devices, skin conditions, or previous abdominal surgery precluded right-sided placement. Glove changes were performed before tunnelling and before handling the shunt, with additional changes when assistance was required at either the cranial or abdominal operative site. A strict separation between the cranial and abdominal operative fields was maintained throughout the procedure.

All patients received a Miethke proGAV 2.0 valve with an adjustable opening pressure setting at 5 and a fixed gravitational setting of 25 cm H_2_O. Antibiotic-impregnated shunt systems were used in the majority of patients, accounting for approximately 88% of cases. All surgeries were performed by the same two senior consultant neurosurgeons (E.E., A.E.T.).

Postoperatively, all patients underwent routine cranial and abdominal CT imaging approximately two hours after surgery without exception to confirm correct shunt placement and exclude bleeding complications. Patients remained fasting until the postoperative imaging had been reviewed and approved by the operating surgeons, allowing immediate revision surgery if suboptimal catheter positioning was identified.

Patients were typically admitted the day prior to surgery, operated on the following day, and discharged on the first postoperative day. Anticoagulation therapy (e.g., direct oral anticoagulants, vitamin K antagonists, etc.) was discontinued one week prior to surgery and resumed three days postoperatively. Follow-up imaging was performed at the referring center approximately 6 to 8 weeks after surgery. Overdrainage was assessed using the follow-up CT scan. Clinical follow-up included a standardized telephone assessment at one month postoperatively. In addition, a written feedback form was distributed at the same time point to capture a range of patient-reported experience measures (PREMs). PREMs were assessed using 3- or 4-point Likert scales, depending on the specific outcome. A likelihood-to-recommend score, reflecting the likelihood of recommending the service on a scale from 1 to 10, was also recorded. Long-term shunt management and valve adjustments were routinely maintained by the referring neurology department. All patients and their outcomes were reported to the Swedish Hydrocephalus registry.

## Data collection and outcomes

Clinical data were prospectively collected for all patients, including demographic characteristics, perioperative variables, and postoperative outcomes up to 30 days postoperatively. All postoperative outcomes were reported by the patients themselves. Computed tomography (CT) imaging was performed for all patients at 6–8 weeks postoperatively. Primary clinical outcomes included complication rates, and reoperation rates during follow-up. Complications were further categorized according to Ibañez, which categorizes based on the need for treatment [16].

### Statistical analysis

Descriptive statistics are reported as means with standard deviations for continuous variables and counts with percentages for categorical variables. Statistical significance was set at a *p*-value < 0.05. Analyses and graph creation were conducted using R software (version 4.2.3, R Foundation for Statistical Computing, Vienna, Austria) [24].

### Ethics and consent

This study received approval from the Swedish Ethical Review Authority (Dnr: 2025–08513-1).

## Results

A total of 80 patients were included between November 2024 and November 2025. All patients (100%) were successfully contacted by telephone for follow-up assessment of patient-reported clinical outcomes. Postoperative questionnaires were distributed to 72 patients, of whom 60 responded (83.3%). The mean time from referral to surgery was 28.5 ± 15 days.

## Clinical outcomes

**Fig. 1:**
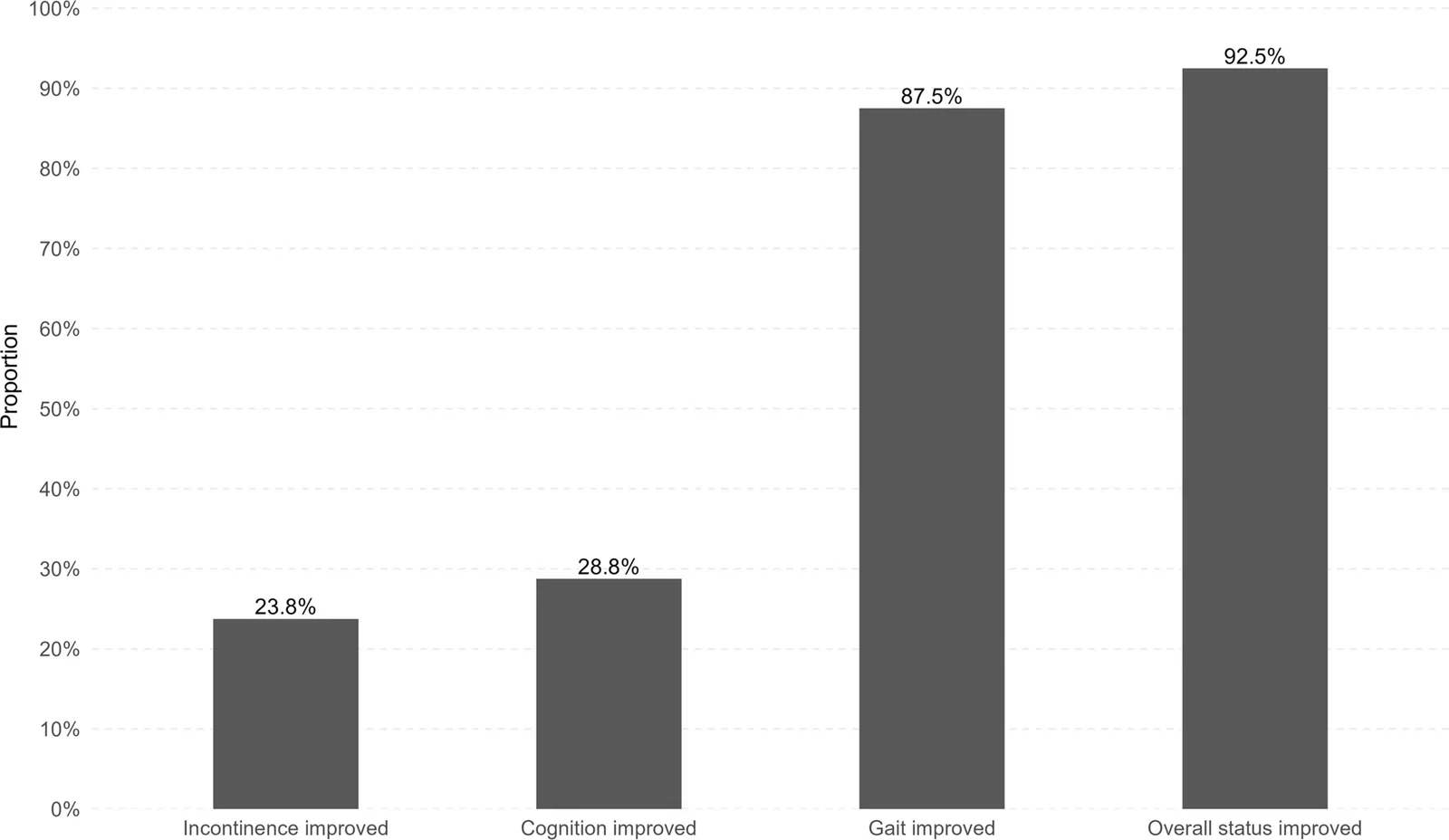
Bar chart of patient-reported clinical improvement one month after ventriculoperitoneal shunt placement (n = 80).

Overall clinical status improved in 74 of 80 patients (93%). Gait improvement was reported in 70 patients (88%), while improvements in urinary continence and cognition were reported in 19 (24%) and 23 patients (29%), respectively (Table 2, Fig. 1). No reoperations were performed within the 30-day study follow-up period. No proximal catheter was misplaced, and all catheters had an acceptable intraventricular placement. No catheter required revision within 30 days. No shunt infections occurred during the 30-day follow-up or during subsequent follow-up. No cases of overdrainage or subdural collections were detected.

## Patient-reported outcome and experience measures

**Fig. 2:**
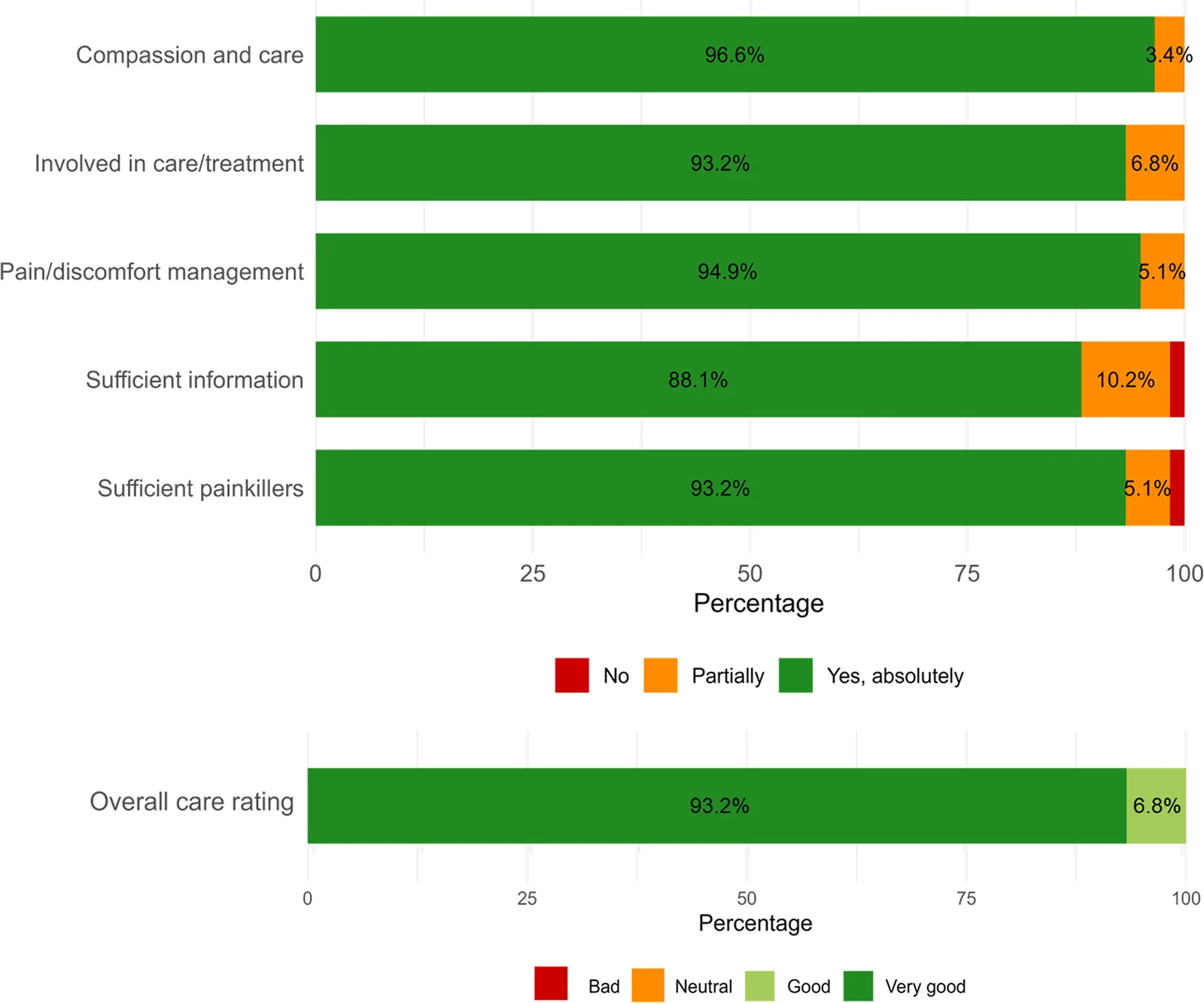
Grotta bar charts of patient-reported experience measures

Among the 60 respondents of the questionnaire, 59 patients (98.3%) reported uneventful wound healing. PREMs, including perceived compassion of care, overall care rating, adequacy of information, involvement in decision-making, and pain management, indicated high overall satisfaction (Table 3, Fig. 2). Two patients reported seeking medical attention for postoperative complaints during the 30-day follow-up period, including a case of headache and a case of persistent dizziness. Reported complications included one grade Ib complication, consisting of a urinary tract infection, and one grade IIb complication consisting of catheter migration to the subcutaneous space. The latter was treated surgically after the study follow-up period. The mean likelihood-to-recommend was 9.8 ± 0.69 (Table 3).

**Table 2:** Clinical effectiveness outcomes following ventriculoperitoneal shunt placement for idiopathic normal pressure hydrocephalus

Variable	Result
Overall status improved, *n* (%)	74 (92.5%)
Gait improved, *n* (%)	70 (87.5%)
Incontinence improved, *n* (%)	19 (23.8%)
Cognition improved, *n* (%)	23 (28.8%)
Referral to operation, mean ± SD days	28.5 ± 15

**Table 3:** Postoperative patient-reported experience measures and clinical outcomes, including wound healing, perceived quality of care, involvement in treatment, and satisfaction with pain management

Variable	Result
Wound healing, *n* (%)	
Uneventful wound healing	59 (98.3%)
Sought medical care for other complications, *n* (%)	2 (3.3%)
Treated with compassion and care, *n* (%)	58 (96.7%)
Yes, absolutely	56 (96.6%)
Partially	2 (3.4%)
No	0 (0%)
Missing, *n* (%)	2 (3.4%)
Overall care rating, *n* (%)	
Very good	55 (93.2%)
Good	4 (6.8%)
Neutral	0 (0%)
Bad	0 (0%)
Missing, *n *(%)	1 (1.7%)
Sufficient information about care/treatment, *n* (%)	
Yes, absolutely	52 (88.1%)
Partially	6 (10.2%)
No	1 (1.7%)
Missing, *n *(%)	1 (1.7%)
Involved in care/treatment, *n* (%)	
Yes, absolutely	55 (93.2%)
Partially	4 (6.8%)
No	0 (0%)
Missing, *n *(%)	1 (1.7%)
Satisfaction with pain/discomfort management, *n* (%)	
Yes, absolutely	56 (94.9%)
Partially	3 (5.1%)
No	0 (0%)
Missing, *n* (%)	1 (1.7%)
Sufficient painkillers, *n* (%)	
Yes, absolutely	55 (91.7%)
Partially	3 (5.0%)
No	1 (1.7%)
Missing, *n *(%)	1 (1.7%)
Likelihood-to-recommend score, mean ± SD	9.8 ± 0.69

## Discussion

Idiopathic NPH has long been underdiagnosed and undertreated, leaving many patients without adequate care and causing substantial burden for both patients and their caregivers [23, 27]. In recent years, however, iNPH has received increasing attention, and more research has been directed toward this condition [11, 18, 25, 31]. Given the aging population and the expected parallel increase in disease prevalence, it is both appropriate and necessary to invest additional resources in the diagnosis and management of iNPH [5].

In contrast, recent national registry data from Sweden demonstrate persistently prolonged waiting times for NPH surgery, with all centers performing below national target benchmarks [28]. The Swedish National Hydrocephalus Registry (NKH) working group has therefore identified reducing waiting times as a priority area. Currently, VP shunt placement is still primarily performed at the neurosurgical departments of university hospitals [28]. At these departments, iNPH surgery commonly falls among lower-priority elective procedures, competing with more complex, time-critical, and resource-intensive intracranial operations [30]. Shunt revisions, along with the placement of external ventricular drains, are common on-call procedures, and consequently these procedures are learned early during neurosurgical residency. Thus, elective shunt placement surgery is commonly performed by residents as part of their neurosurgical training [26]. Arguably, this may place iNPH patients at a disadvantage within university hospital systems, given evidence that limited surgeon experience is an independent predictor of higher rates of complications, shunt infection, shunt revision, and shunt failure [2, 8, 22, 29]. To provide a scalable alternative, a dedicated iNPH center was established at a clinic specialized in elective degenerative spine surgery. Private healthcare clinics operating under public contracts for degenerative spinal conditions have a long-standing history of successful implementation in Sweden and currently perform the overwhelming majority of these procedures nationwide.

The iNPH center was established using care bundle principles after securing the necessary contracts to accept referrals and treat iNPH [14, 21]. The care bundle strategy includes clarifying the current evidence for best clinical practice along with the most important problems to solve and defining a limited number of key metrics to assess the most relevant outcomes. To improve these outcomes, a dedicated team was assembled and educated, and performance was regularly evaluated and discussed to facilitate continuous refinement of the care pathway. All patients were managed by two senior neurosurgical consultants with more than 20 years of experience. The care bundle further incorporated standardized patient selection, preoperative assessment, surgical technique, postoperative imaging, and follow-up pathways, thereby aiming to improve consistency of care across the entire patient journey. Unlike prior care bundles for shunt surgery, which have focused narrowly on shunt infection prevention, the present bundle addressed risk across the entire pathway, from referral to postoperative follow-up [10, 14, 21].

In this study, we found that clinically relevant outcomes improved substantially following VP shunt implantation. Two patients reported complications, both of which were classed as minor. Furthermore, patient-reported experience measures (PREM) demonstrated a high level of satisfaction across the cohort. While the present study was not designed to determine the contribution of individual bundle components, the favorable outcomes observed likely reflect the combined effect of the consultant-led care bundle strategy rather than any single intervention.

An important achievement was the short referral-to-surgery interval, with a mean waiting time of 28.5 days. Most patients were referrals from neurosurgical departments where surgical waiting times exceeded the stipulated national maximum of 90 days. However, by the patients, the time passed since symptom onset was often reported as more than a year, reflecting the many instances of delays, as the NPH management pathway includes a referral from a general practitioner (GP) to a neurologist and subsequently to a neurosurgeon if the patient is deemed a candidate for shunt treatment. Thus, minimizing further delays was prioritized, and most surgeries were performed within one month of referral. This contrasts with the afore-mentioned Swedish national registry data demonstrating persistently prolonged waiting times in all parts of the NPH management pathway. Data are compared to the stipulated 90 + 90 days (26 weeks) from referral to meeting a neurologist and surgical waiting time after meeting a neurosurgeon. All centers reported median waiting times at or beyond 34 weeks, and two out of six regional centers reported more than 70 weeks [28]. At the center where waiting times were the lowest, only 31% of patients were managed within 90 + 90 days. The finding from this report is particularly relevant, as shorter waiting times from surgical indication to intervention (≤ 3 months) have been associated with improved outcomes in NPH [7].

When examining patient-reported clinical outcomes, VP shunt placement demonstrated high efficacy, particularly regarding overall well-being and gait improvement. Improvements in continence and cognition were less pronounced. While previous meta-analyses have also reported lower rates of improvement in these domains, the discrepancy observed in our study is more substantial [11]. Specifically, a meta-analysis by Giordan et al. demonstrated that 75% of patients experienced gait improvement, more than 60% experienced improvement in cognitive function, and 55% experienced improvement in continence. In contrast, our results were 88%, 29%, and 24%, respectively. This discrepancy is consistent with established iNPH literature, which has reported greater improvement in gait than in cognitive and urinary symptoms following shunt placement [11]. Only patient-reported outcomes were available at this early timepoint, and the one-month interval may underestimate certain improvements in domains that have been shown to respond more gradually to shunting. This may be explained by the self-reported nature of the outcomes, especially given that data were collected via telephone, where biases such as recall bias and hindsight bias may play a role. Nonetheless, both PREMs and clinical outcome measures indicate that patients were overall very satisfied with their treatment and would recommend it to others.

Regarding safety, we observed low reoperation rates, which compare favorably to revision rates of approximately 16% reported in meta-analyses [11, 31]. When considering complication rates, approximately 3.3% of respondents reported complications. According to the Ibañez classification, these events were graded as Ib and IIb, respectively, indicating relatively minor complications [16]. These findings suggest that, in our cohort of patients treated by experienced consultants, complication rates were low and may compare favorably with those reported in studies predominantly conducted at university hospitals. Whether this is attributable to surgeon experience, the care bundle strategy, patient selection, or a combination of these factors cannot be determined from the present study. Thus, the data underscore the feasibility of performing VP shunt surgery in a non-academic private setting, thereby potentially increasing access to care.

## Limitations

This study has several limitations. First, the retrospective design introduces potential biases that may affect the results and their interpretation. Second, the sample size, while reasonable for a single-center study, remains relatively small compared to larger NPH cohorts. Third, outcomes were primarily self-reported, making them inherently subjective and more susceptible to bias, despite efforts to standardize data collection. Fourth, the 30-day follow-up period captures acute perioperative morbidity and placement accuracy, but does not capture long-term shunt failure, delayed infections, or late revisions. Finally, because multiple components of the care bundle were implemented simultaneously, the relative contribution of individual bundle elements to the observed outcomes could not be determined. Additionally, patients with significant comorbidities were excluded from treatment, which likely contributed to the low complication rate and limits generalizability to iNPH populations with higher risks of complications, such as those typically seen at university hospitals.

## Conclusion

A dedicated care bundle strategy for iNPH at a private surgical center was associated with short referral-to-surgery times, high patient satisfaction, substantial patient-reported clinical improvement, and low complication and reoperation rates. These findings suggest that a structured consultant-led care bundle can support safe and effective VP shunt surgery outside university hospital settings. Wider implementation of dedicated iNPH care bundle pathways may help reduce treatment delays and improve timely access to care.

## Data Availability

The data in support of our findings can be obtained upon reasonable request from the corresponding author.
